# The Relationship Between MR Demonstration of Extramural Venous Invasion and Nodal Disease in Rectal Cancer

**DOI:** 10.4137/cmo.s370

**Published:** 2008-04-01

**Authors:** Dow-Mu Koh, Neil J. Smith, R. Ian Swift, Gina Brown

**Affiliations:** 1 Academic Department of Radiology, Royal Marsden Hospital, U.K.; 2 Department of Colorectal Surgery, Mayday University Hospital, U.K

**Keywords:** MR imaging, node, staging, rectal cancer

## Abstract

**Purpose:**

To investigate the relationship between extramural venous invasion (EMVI) detected at T2-weighted MRI and nodal disease rectal cancer compared with histopathology.

**Materials and Methods:**

The MR imaging of 79 consecutive patients with rectal cancer who underwent primary rectal surgery without neoadjuvant treatment were reviewed. MR images were scored by an expert radiologist for the presence and degree of EMVI using a five point scale blinded to pathological findings. Receiver operating characteristic curve analyses were performed to determine the sensitivity and specificity of MRI scoring in predicting EMVI and nodal disease at histopathology.

**Results:**

Compared with histology, an MR score of >2 was found to have 100% sensitivity (95% CI: 77%–100%) and 89% specificity (95% CI: 79%–96%) in identifying EMVI involving veins >3 mm in diameter. An EMVI score of >2 was had a sensitivity of 56% (95% CI: 30%–80%) and specificity of 81% (95% CI: 69%–90%) for identifying patients with stage N2 disease.

**Conclusions:**

EMVI score of >2 on T2-weighted MR imaging has a high sensitivity and specificity for histopathologically proven extramural venous invasion involving venules ≥3 mm in diameter. However, EMVI scores have only moderate sensitivity in the predicting nodal involvement.

## Introduction

Thin section T2-weighted magnetic resonance imaging (MRI) can be used to demonstrate a number of adverse prognostic features for local disease recurrence and survival in patients with rectal cancer (1), which were initially established from histopathological studies (1, 2). These include tumour extension to the lateral resection margin, the depth of extramural tumour extension and the presence of extramural venous invasion (EMVI).

The normal outer rectal wall is perforated by numerous small venules, which appear as low to intermediate signal intensity tubular structures at T2-weighted MR imaging (3). Extramural venous invasion is recognised at T2-weighted MR imaging by the expansion and irregularity of these venules adjacent to the primary rectal tumour, due to contiguous tumour extension. The involved vein usually appears intermediate signal intensity with loss of the normal vascular flow void (1). Previous study has demonstrated good agreement (kappa = 0.64) between MR detection of extramural venous invasion and the presence of tumour extension into extramural veins measuring 3 mm or greater in diameter at histopathology (1). However, extramural tumour extension into small venules (<3 mm in diameter) can still be beyond the spatial resolution of current MR imaging techniques to confidently detect.

It is well recognised that lymphatic channels accompany or parallel the course of blood vessels in the body (4). However, lymphatic vessels are smaller than blood vessels and as such, normal mesorectal lymphatics are not directly visualised at T2-weighted MR imaging. Nevertheless, we hypothesise that MR evidence of tumour invasion into extramural veins may act as a surrogate marker for lymphatic permeation by tumour. Hence, patients with MR evidence of extramural venous invasion would theoretically carry a higher risk and incidence of nodal disease. However, the potential relationship between MRI determined extramural venous invasion and the presence of nodal disease has not been previously investigated.

From a practical point of view, the recognition of MR imaging features that are associated with increased risk of nodal disease can alert the radiologist to carefully scrutinise the staging examination for nodal disease. An association between extramural venous invasion visualised at MRI and nodal disease, particularly stage N2 nodal involvement, can also strengthen the argument for neoadjuvant chemoradiation in patients demonstrating such features, with the aim of reducing local disease recurrence.

Hence, the aim of this study was to investigate the relationship between EMVI detected at T2-weighted MRI and nodal disease in patients with rectal carcinoma compared with histopathology.

## Materials and Methods

Approval from the institutional research review board was obtained for retrospective analysis of database.

### Patient population

The clinical and pathological details of consecutive patients undergoing surgical resection of a primary colorectal cancer between January, 2000 and December 2004 at a single hospital were compiled on a database. From these, seventy-nine consecutive patients with rectal cancer were identified and analysed. The inclusion criteria were: (a) Patients had biopsy proven rectal carcinoma, (b) the patients were selected to be treated by primary surgery without pre-operative chemoradiation through a multidisciplinary meeting based on MRI and clinical findings, (c) patients underwent MRI staging prior to surgery and (d) the pathological records and material from the tumour were available for review. Patients with contraindications to MR imaging were excluded.

### MR imaging studies

MRI was performed using thin section (3 mm) high spatial resolution T2-weighted technique on 1.5T MR systems (Siemens’ Vision, Erlangen, Germany; Philip’s Intera, Best, The Nederlands) prior to surgery.

At each study, T2-weighted sagittal images (TR > 5000 ms, TE = 128 ms, ETL = 16, 3 mm thickness, 350 cm FOV, 512 × 512 matrix, NEX = 3, scan duration = 4 min) of the rectum were first obtained to enable planning of the axial sections. Contiguous T2-weighted axial 3 mm images of the pelvis (TR > 5000 ms, TE = 128 ms, ETL = 16, 3 mm thickness, 185 mm FOV, 256 × 256 matrix, NEX = 3, scan duration = 3–12 min) were acquired in a plane perpendicular to the rectal wall, from the anorectal junction along the length of the mesorectum. Coronal imaging was performed parallel to the anal canal using similar scan parameters as the axial imaging.

### Image interpretation

The in-vivo MR images were reviewed by an expert radiologist, who has more than 10 years experience in interpreting rectal MR images using an image workstation (efilm workstation, version 2.0; Merge Healthcare, Milwaukee) blinded to the results of histopathology.

On imaging, a tumour was classified as a low rectal tumour if the bulk of the tumour was below the level of the origin of the levator ani muscle at the pelvic sidewall. A mid-rectal tumour was located above the levator origin but below the anterior peritoneal reflection. An upper rectal tumour was defined as arising at or above the anterior peritoneal reflection.

**Figure 1:**
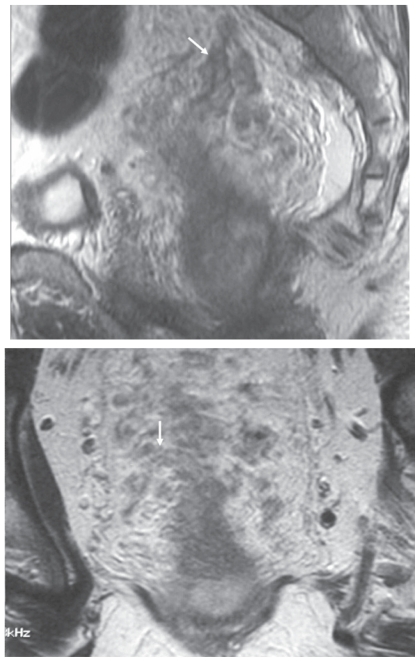
MR imaging demonstration of extramural venous invasion. T2-weighted imaging in the sagittal and coronal planes demonstrating multiple expanded serpingenous structures (arrows) of intermediate signal intensity emanating from the tumour. This is an example of grade 4 extramural vascular invasion.

**Figure 2:**
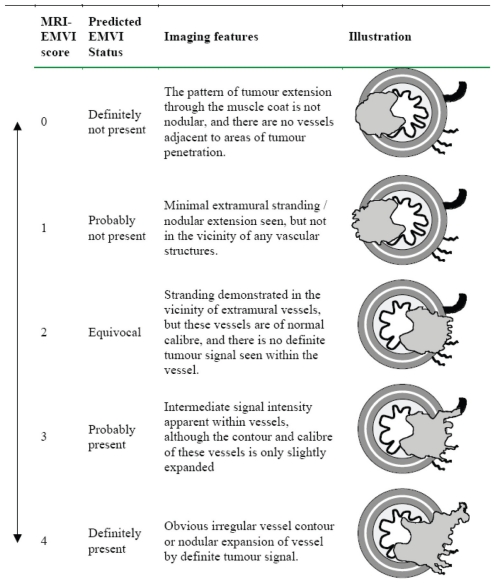
Five-point scoring system for MRI detected extramural venous invasion.

In each patient, the presence of extramural venous invasion was identified by intermediate signal intensity tumour extending within and along veins lying adjacent to and contiguous with the primary tumour on T2-weighted MR imaging (Fig. 1). The involved veins may be expanded or show irregularity of the venous contours. The presence and degree of EMV was graded using a five point scale which ranged from 0 to 4 (Fig. 2).

### Comparison with histopathology

Following total mesorectal excision surgery, the surgical specimen was fixed in formalin and the pathological examination performed according to National guidelines (2). The reporting was completed using proformas compliant with the National minimum dataset by one of three consultant histopathologists (with over 10 years experience). The presence of EMVI was assessed using a standard definition: the presence of a rounded mass of tumour tissue within an endothelium-lined space, which was either surrounded by a rim of smooth muscle or contained red blood cells. The presence or absence of EMVI was recorded as being EMVI positive or EMVI negative. In patients who were EMVI positive, note was also made of whether the EMVI by tumour involved small (<3 mm in diameter) or large (≥3 mm in diameter) veins.

Inaddition, the mesorectal surgical specimen was evaluated for the nodal status. The nodal stage was determined on a per patient basis according to the TNM classification system (5). A node-by-node comparison between MR imaging and histopathology was not undertaken as part of this study.

### Statistical analysis

Statistical analysis was performed using Medcalc for Windows statistical software Version 8.1.1.0 (MedCalc Software, Mariakerke, Belgium). Receiver operating curve (ROC) was performed to determine the sensitivity and specificity of MR scoring in identifying EMVI involvement of veins ≥3 mm in diameter at histopathology.

Receivers operating characteristic (ROC) curves analysis was also performed to determine if the sensitivity and specificity of EMVI grading in (a) distinguishing patients with no nodal disease (stage N0) from those with nodal disease (stage N1 or N2) and (b) distinguishing patients with good prognosis stage N0 or N1 nodal disease from those with poor prognosis stage N2 nodal disease.

## Results

There were 42 males and 37 females with a mean age of 67.8 years (range: 40 to 86 years). 18/79 (22.8%) of tumours were located in the lower rectum, 26/79 (32.9%) in the mid-rectum, 21/79 (26.6%) in the upper rectum and 14/79 (17.7%) were at the rectosigmoid.

The tumour stages by histopathology were as follows: 1/79 (1.3%) T1, 28/79 (35.4%) T2, 40/79 (50.6%) T3 and 10/79 (12.7%) T4. The T3 tumours were early stage with <5 mm maximum extramural tumour depth, and stage T4 resulted from peritoneal infiltration by high rectosigmoid tumours. At histopathology, the nodal status was found to be as follows: 45/79 (57%) had stage N0 disease, 18/79 (22.8%) had stage N1 disease and 16/79 (20.3%) had stage N2 disease.

### Comparison of MR grading of EMVI with histopathology

33/79 (42%) of patients demonstrated EMVI of grades 1 or greater at T2-weighted MR imaging. The distribution of EMVI grading as determined by MR imaging in our study population is summarised in Table 1.

At histopathology, 58/79 (73.4%) of the patients had no evidence of EMVI. Of the 21/79 (26.8%) that showed EMVI at histopathology, 7/79 (8.9%) demonstrated EMVI involving only small venules less than 3 mm in diameter. 14/79 (17.9%) showed EMVI involving venules 3mm or greater in diameter.

**Figure 3:**
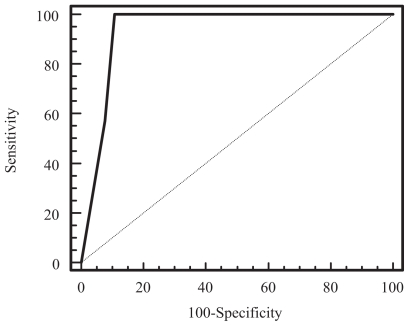
Receiver operating characteristic curve of the MR grading of extramural venous invasion versus the presence of extramural venous invasion involving venules 3 mm of greater in diameter at histopathology (Az = 0.94).

A receiver operating curve analysis was performed to determine the relationship of the MR grading of EMVI versus the absence or presence of histologically proven EMV involving venules 3 mm or greater in diameter. The ROC curve is shown in Figure 3. An MR score of more than 2 was found to have 100% sensitivity (95% CI: 77%–100%) and 89% specificity (95% CI: 79%–96%) in identifying the presence of extramural venous invasion involving veins 3 mm or greater in diameter.

### The relationship between MR determined extramural venous invasion and nodal disease

**Figure 4:**
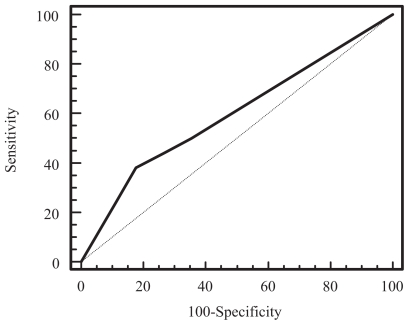
Receiver operating characteristic curve of MR grading of extramural venous invasion for identifying patients with stage N1 or N2 disease from those with N0 disease (Az = 0.59).

The receiver operating curve of MR grading of EMVI for identifying patients with no nodal disease (stage N0) versus those with nodal disease (stage N1 or N2) was as shown in Figure 4. The area under the curve (Az) was 0.59. An EMV score of greater than 2 had a sensitivity of 38% (95% CI: 22%–56%) and specificity of 82% (95% CI: 68%–92%) for identifying patients with either stage N1 or N2 disease.

**Figure 5:**
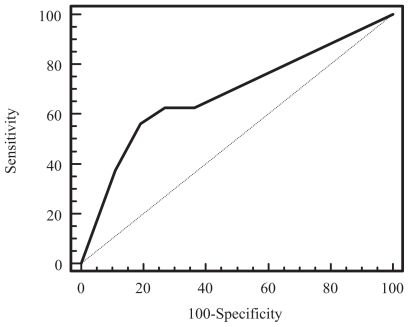
Receiver operating characteristic curve of MR grading of extramural venous invasion for identifying patients with stage N2 disease from those with N1 or N0 disease (Az = 0.68).

By comparison, the receiver operating curve of MR grading of EMVI for identifying patients with stage N0 or N1 disease versus those with stage N2 nodal disease is as shown in Figure 5. The area under the curve (Az) was 0.68. An EMVI score of greater than 2 was found to have a sensitivity of 56% (95% CI: 30%–80%) and specificity of 81% (95% CI: 69%–90%) for identifying patients with stage N2 disease.

## Discussion

Pathologically proven extramural venous invasion (EMVI) in rectal cancer has been shown to be associated with poorer survival (6), increased risk of nodal disease (7, 8) and distant metastases (9). However, the definitive identification of EMVI currently relies on pathological examination following surgical resection. The prospective identification of EMVI prior to surgery using non-invasive MRI is desirable since confident identification of patients with EMVI prior to surgery may prompt the use of neoadjuvant chemoradiation treatment, with the aim of reducing long term local and systemic disease recurrence.

Extramural venous invasion can be identified in-vivo using thin section (3 mm) high spatial resolution T2-weighted MRI (1). A recent study demonstrated that using such a technique, MRI has an overall sensitivity of 62% and specificity of 88% in detecting EMVI compared with histopathology. In this study, we found that an EMVI grading score of more than two on MRI had 100% sensitivity and 89% specificity in identifying EMVI involving veins 3 mm of greater than diameter. It was perhaps not surprising that MR imaging was successful in characterising the involvement of larger venules (3), however, involvement of small venules may still be beyond the spatial resolution of the current MR imaging technique to detect.

The grading of EMVI in this study is based on observed patterns of extramural infiltration of tumour at T2-weighted MR imaging. The outer longitudinal muscle layer of the rectal wall is normally perforated at numerous points by small venules (3). The invasive edge of a tumour can infiltrate through the outer longitudinal muscle layer, to grow along and within these venules. This venous or peri-venular tumour growth appears isointense to the primary tumour, and results in loss of signal flow void of the blood vessel (3). With increasing infiltration, there is expansion and irregular beading along the length of the involved vein. The extramural venous extension can grow over considerable distances, and can even extend along the superior rectal veins. Extramural venous invasion can affect single or multiple venules adjacent to the primary tumour.

Although advanced EMVI can be detected with reasonable confidence at MRI, it is unclear if such an observation may be used as a surrogate indicator of the likelihood or degree of nodal involvement. Although lymphatic invasion has been shown to be associated with an increased risk of nodal disease (8), this may be difficult to identify even at histopathology. However, because of the close anatomical relationship between venous and lymphatic vessels, we hypothesised that EMVI may be a surrogate sign for lymphatic invasion and hence, an increased likelihood of nodal disease when EMVI is present. There is also some evidence to suggest that venous invasion itself increases the likelihood of nodal disease. It has been shown in patients with rectal cancer confined to the rectal wall that intramural venous invasion was significantly associated with nodal disease (10), suggesting that venous invasion may be an independent mechanism for nodal spread of tumour. Extramural venous invasion represents a form extramural tumour extension, and it is also well recognised that the incidence of nodal involvement in patients with rectal cancer increases with the degree of tumour extension beyond the rectal wall (11).

In our study, we examined the relationship between EMVI and nodal disease. By applying ROC analysis, an EMVI grading of greater than 2 was found to have a moderate sensitivity (56%) but high specificity (81%) for discriminating between patients with stage N2 disease those with stage N0 or N1 disease. Although the sensitivity of using EMVI to identify stage N2 nodal disease is at best moderate, the presence of EMVI may help to alert radiologists to carefully scrutinise the MR images for evidence of nodal disease in patients to improve malignant nodal detection.

The low sensitivity for EMVI as an overall predictor of nodal disease (stage N1 or N2) suggests that nodal metastasis in rectal cancer is a complex process. Clearly, a cluster of invasive tumour cells may penetrate lymphatics or vascular channels to be translocated to the lymph node without demonstrating contiguous tumour growth along the vessels. Metastases may also arise from vascular or lymphatic invasion within the rectal wall by budding and dividing tumour cells (12), which cannot be resolved by MR imaging.

There were a few limitations to this study. Firstly, the MR grading of EMVI was not directly compared with histopathology. Nevertheless, the ROC analysis suggested a high sensitivity and specificity of EMVI grades of greater than 2 for the presence of pathologically proven extramural venous tumour invasion of veins larger than 3 mm in diameter. Clearly, future work to enable direct radiological-pathological comparison of the MR imaging grading of EMVI with histology would be welcomed. Secondly, node-by-node radiological-pathological comparison was not undertaken in this study. However, we were interested in comparing the MR imaging grading of EMVI with the pathological nodal staging on a per patient basis, and node-by-node correlation in this case was not necessary.

## Conclusions

A grading of EMVI of greater than 2 on T2-weighted MR imaging has a high sensitivity and specificity for histopathologically proven extramural venous invasion involving venules ≥ 3 mm in diameter. An EMVI scoring of greater than 2 had a high specificity for nodal disease, but only a moderate sensitivity in the detection of nodal involvement and poor prognosis stage N2 disease. This suggests that nodal metastasis is a complex process and extramural venous invasion represents only one possible mechanism of its pathogenesis.

## Figures and Tables

**Table 1:** Grading and frequency of extramural venous invasion (EVM) in patients with rectal carcinoma (n = 79).

EVM grade	Number of cases (%)
0	46 (58.2%)
1	6 (7.6%)
2	6 (7.6%)
3	8 (10.1%)
4	13 (16.5%)
